# Hyponatremia Correction Rates and Mortality: Causality or Epiphenomenon?

**DOI:** 10.34067/KID.0000000000000414

**Published:** 2024-03-13

**Authors:** Helbert Rondon-Berrios, Richard H. Sterns

**Affiliations:** 1 Renal-Electrolyte Division, University of Pittsburgh School of Medicine, Pittsburgh, Pennsylvania; 2 University of Rochester School of Medicine and Dentistry, Rochester, New York

**Keywords:** hyponatremia

## Introduction

Rapid correction of chronic hyponatremia has long been associated with neurologic complications, namely the osmotic demyelination syndrome. However, for nearly 40 years, some authors have denied the validity of the association, claiming not only the lack of harm from rapid correction but also a mortality benefit.^1,2^

The idea that rapid correction of hyponatremia reduces mortality was recently supported by two large retrospective studies, one derived from a large multicenter public database of over 200,000 unique intensive care unit patients at 208 hospitals across the United States^3^ and one in a cohort of 3274 patients with a plasma sodium concentration (PNa) <120 mmol/L admitted to two large academic hospitals in Boston over the course of 15 years.^4^ Details of these two studies and others that address the statistical association between PNa correction rates and mortality are summarized in Table 1.

**Table 1:** Studies examining the statistical association between hyponatremia correction rates and mortality outcomes

Author	Year	Sample Size	PNa Cutoff Inclusion Criteria, mmol/L	Mean PNa±SD, mmol/L	PNa Correction Rates	Mortality Based on Correction Rates	Causes of Death	Association Between Correction Rates and Mortality	Major Limitations
Kang *et al.*^5^	2012	116	<120	114.9±5.2	<6 versus ≥6 mmol/L per 24 h	In-hospital mortality rates of 43.9% and 13.4% for <6 and ≥6 mmol/L per 24 h, respectively	Malignancy (8.6%), pneumonia (3.4%), and hepatic failure (1.7%)	Correction <6 mmol/L per 24 h is associated with increased mortality (RR, 2.652; 95% CI, 1.081 to 6.507; *P* = 0.033)	High proportion of patients with kidney disease (20.7%), metastatic cancer (19.8%), and liver disease (12.9%)
Geoghegan *et al.*^6^	2015	412	<120	117^a^	≤5 (undercorrection) versus 6–10 (optimal) versus >10 mmol/L per 24 h (overcorrection)	In-hospital mortality rates of 12%, 5%, and 5% for undercorrection, optimal, and overcorrection, respectively	Not reported	Undercorrection and overcorrection were not associated with increased mortality (OR, 2.2; 95% CI, 0.8 to 5.6 and OR, 1; 95% CI, 0.4 to 3.1, respectively)	Undercorrection group had the highest CCI scores and higher proportion of heart failure, cirrhosis, or severe kidney disease
Krummel *et al.*^7^	2016	147	<120	115±4.7^b^ 121±10.4^c^	Excessive correction: ≥12 mmol/L per 24 h or ≥18 mmol/L per 48 h	Not reported	Not reported	Excessive correction was not associated with increased mortality (HR, 0.76; 95% CI, 0.33 to 1.77; *P* = 0.52)	Patients with PNa <110 mmol/L had the lowest CCI scores and the lowest mortality
Turkmen *et al.*^8^	2022	145	<115	110^a^	<6 versus 6–10 versus >10 mmol/L per 24 h	In-hospital mortality rates of 26.5%, 9.4%, and 6.9% for <6, 6–10, >10 mmol/L per 24 h, respectively	Not reported	Correction <6 mmol/L per 24 h was associated with increased mortality (OR, 6.68; 95% CI, 2.00 to 22.32; *P* = 0.002)	Do not report baseline comorbidities or summary comorbidity measures based on correction rates Do not report variables used in logistic regression
Kinoshita *et al.*^3^	2023	1024	<120	116.37±9.49^d^ 117.02±6.37^e^	≤8 versus >8 mmol/L per 24 h	In-hospital mortality rates of 13.4% and 8.4% for ≤8 and >8 mmol/L per 24 h, respectively	Not reported	In-hospital mortality was significantly lower in rapid correction group (absolute difference, −4.37%; 95% CI, −8.47% to −0.26%). No association was found using two other models (g-formula and PS adjustment), neither in a secondary analysis using trichotomous rates of correction (≤8, 9–12, and >12 mmol/L per 24 h)	<8 mmol/L per 24 h group had a higher proportion of patients with congestive heart failure (22% versus 14%), chronic kidney disease (13.1% versus 5.3%), and liver failure (4.5% versus 2.9%) compared with the >8 mmol per 24 h group
Seethapathy *et al.*^4^	2023	3274	<120	116±4	<6 versus 6–10 versus >10 mmol/L per 24 h	In-hospital mortality rates of 13%, 8%, and 5% and 30-d mortality rates of 21%, 11% and 8% for <6, 6–10 and >10 mmol/L per 24 h, respectively	Cancer (33%), infection (25%), cardiovascular disease (14%), and liver disease (10%). Documented cerebral edema directly contributed to the death of single patient, a marathon runner with acute hyponatremia	Correction <6 mmol/L per 24 h was associated with increased in-hospital mortality and 30-d mortality (OR, 1.71; 95% CI, 1.27 to 2.31 and OR, 2.13; 95% CI, 1.64 to 2.77, respectively). Correction >10 mmol/L per 24 h was associated with decreased in-hospital and 30-d mortality (OR, 0.64; 95% CI, 0.44 to 0.93 and OR, 0.69; 95% CI, 0.50 to 0.96, respectively). PS-weighted analysis showed no mortality benefit with correction >10 mmol/L per 24 h. In subgroup analysis, correction <6 mmol/L per 24 h in patients with heart failure and cancer was associated with increased mortality, while correction >10 mmol/L per 24 h in patients with cirrhosis was associated with decreased mortality	<6 mmol/L per 24 h group had the highest CCI scores and highest proportion of patients with cirrhosis, heart failure, and metastatic cancer, while patients in >10 mmol/L per 24 h group had the lowest CCI scores and the highest proportion of patients with schizophrenia. Only 12% of patients were treated with HTS. Subgroup analysis did not account for severity of disease

CCI, Charlson comorbidity index; CI, confidence interval; HR, hazard ratio; HTS, hypertonic saline; OR, odds ratio; PNa, plasma sodium concentration; PS, propensity scores; RR, relative risk.

a Median.

b Nadir.

c On admission.

d Slow correction group.

e Rapid correction group.

### Limitations of Current Studies

Observational studies play a well-established role in clinical research and can potentially provide valuable information to guide clinical practice, particularly in the field of hyponatremia where randomized controlled trials are lacking. However, these studies are often susceptible to flaws that can negatively affect data interpretation and validity of findings. Therefore, it is crucial to critically appraise observational studies and have a clear understanding of these potential flaws. One persistent challenge in observational designs is the need to eliminate confounding. Confounding arises when a seemingly causal relationship between an exposure and an outcome is distorted by the direct or indirect influence of a third variable, known as the confounder.^9^

In the case of hyponatremia, certain comorbidities and their severity (confounders) can influence both PNa correction rates (the exposure) and mortality (the outcome). Advanced stages of cirrhosis, heart failure, and cancer are associated with increased risk of mortality. In addition, these conditions are characterized by sustained arginine vasopressin release and decreased kidney function which impair the ability to excrete large volumes of diluted urine. Unlike patients with self-induced acute water intoxication or chronic hyponatremia due to hypovolemia or medications (conditions that have a favorable prognosis), patients with an impaired ability to excrete dilute urine due to advanced cirrhosis, heart failure, or cancer (conditions with high in-hospital and 30-day mortality rates) are unlikely to experience inadvertent rapid correction of hyponatremia due to a spontaneous water diuresis.

In most studies, patients with slow PNa correction rates had a higher comorbidity burden and a higher proportion of cirrhosis, heart failure, and cancer compared with patients with rapid PNa correction rates. To control for these confounders, many investigators considered summary comorbidity measures (Charlson Comorbidity Index and Acute Physiology, Age, Chronic Health Evaluation IV) and/or made statistical adjustments, such as multivariable logistic regression analysis and propensity score (PS) methods.^3,4^ PS methods are more likely to achieve a similar distribution of observed baseline variables across patients with slow and rapid PNa correction compared with traditional multivariable analysis, and they closely mimic what would be expected in an randomized controlled trial. When PS methods were used, no statistical association between rapid PNa correction and reduced mortality was found.^3,4^ In fact, in one study with PS matching, neurologic outcomes were actually worse with rapid correction.^3^

Subgroup analyses of patients with cirrhosis, heart failure, and cancer have been performed in an effort to make the etiology and chronicity of patients within each comorbidity group more homogeneous. Using multivariable analysis, investigators found a decrease in 30-day mortality with rapid PNa correction rates in a subgroup of patients with cirrhosis while observing increased in-hospital and 30-day mortality with slow PNa correction rates in subgroups of patients with heart failure and cancer.^4^ However, no adjustment for severity of disease within groups was performed. Patients with cirrhosis, heart failure, or cancer of mild severity behave fundamentally differently from patients with advanced forms of these disorders. Patients with mild disease have a better prognosis, and hyponatremia is usually due to a reversible condition, leading to faster rates of correction when the cause of hyponatremia is removed.

The effect of comorbidity adjustments is limited to known (and measured) confounding factors. However, many potential confounders are not always measured. Potential confounders not addressed in these studies are confounding by indication and confounding by frailty.^9^ Confounding by indication means that clinicians are more likely to follow good medical practice and correct PNa slowly in patients with certain comorbidities of great severity (*e.g*., decompensated cirrhosis) that carry a higher risk of neurologic sequelae (osmotic demyelination syndrome) when corrected rapidly. Clinicians' perception is a variable not measured in these studies. Confounding by frailty means that clinicians are more likely to withhold aggressive PNa correction in debilitated patients with comorbidities of great severity (*e.g*., decompensated cirrhosis, decompensated heart failure, and metastatic cancer) who are close to death. Frailty is another variable not measured in these studies.

If PNa correction rates were indeed causally linked to mortality, it would be expected that patients who died with slow PNa correction rates would experience a high prevalence of cerebral edema. However, imaging evidence of cerebral edema was not a measured outcome in these studies, and death from herniation was reported extremely rarely.^4^ Furthermore, if death was caused by hyponatremia itself, one would anticipate that deceased patients would be severely hyponatremic at the time of death. Yet most studies indicate that at the time of death, PNa was either normal or near normal.^4^ Finally, it would be expected that mortality rates would increase as biochemical severity of hyponatremia worsens. However, surprisingly, several studies have shown that once sodium levels drop below 120 mmol/L, there is no increase in mortality despite the worsening hyponatremia; instead mortality actually decreases.^10^ One possible explanation for this observation is that patients who survive with very low PNa (<110 mmol/L) are usually hospitalized due to drug-induced hyponatremia rather than severe illness. On the other hand, hyponatremia of mild to moderate severity (≥120 mmol/L) tends to occur more frequently among patients admitted for life-threatening conditions, such as heart failure or cirrhosis.

It is challenging to discern whether the association between hyponatremia correction rates and mortality observed in some of these studies is the result of clinician actions (such as actively treating hyponatremia) or of inherent characteristic of the patients themselves, such as the nature of the underlying comorbidities and their severity. For example, in one of the retrospective studies,^4^ only 12% of patients were treated with hypertonic saline, suggesting that in most patients whose PNa increased by >10 mmol/L per 24 hours, the cause of the increase was excretion of dilute urine rather than an active attempt by caregivers to correct hyponatremia rapidly.

We eagerly await the results of the *Targeted Correction of Plasma Sodium Levels in Hospitalized Patients with Hyponatremia* (HIT) trial, an international multicenter randomized, controlled trial aiming to enroll 2278 hospitalized adult patients with PNa <130 mmol/L.^10^ This study will compare mortality and 30-day rehospitalization in patients randomized to standard of care or targeted correction of hyponatremia following correction goals and limits recommended by the European clinical practice guidelines. However, the HIT trial may not be large enough to completely resolve the fast/slow correction debate. Documented cerebral edema due to hyponatremia is rarely found in cohort studies^4^; an extraordinarily large study would be required to support or refute the hypothesis that correction >10 mmol/L per 24 hours is necessary to prevent death from herniation.

**Figure 1:**
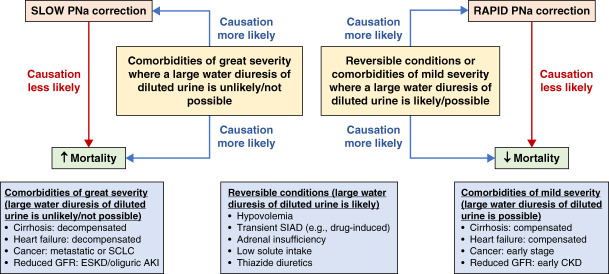
**Explaining the observed association of hyponatremia correction rates and mortality in some studies.** Differences in PNa correction rates of a few mmol/L are less likely to directly influence mortality outcomes in patients with hyponatremia. Instead, it is more probable that both mortality and PNa correction rates are affected by comorbidities and their severity. Patients with hyponatremia resulting from comorbidities of great severity not only face a heightened risk of death but are also unlikely to generate significant volumes of diluted urine (and hence slower PNa correction rates) due to sustained appropriate (*e.g*., decompensated heart failure and cirrhosis) or inappropriate (*e.g*., metastatic cancer or SCLC) AVP secretion or severely reduced GFR (*e.g*., oliguric AKI or ESKD). On the contrary, patients with hyponatremia resulting from comorbidities of mild severity (*e.g*., compensated heart failure or cirrhosis) or reversible conditions (*e.g*., hypovolemia, transient SIAD, adrenal insufficiency, low solute intake, or thiazide diuretics) have a more favorable prognosis. They are also more likely to produce substantial volumes of diluted urine, leading to faster PNa correction rates. This is attributed to the absence of sustained AVP release or the resolution of other factors that hinder urinary dilution. ↑, Increased; ↓, Decreased; AVP, arginine vasopressin; PNa, plasma sodium concentration; SCLC, small-cell lung cancer; SIAD, syndrome of inappropriate antidiuresis.

In conclusion, we believe it is implausible that differences of a few mmol/L in correction rates are responsible for most fatalities associated with hyponatremia. It is far more likely that underlying comorbidities and their severity explain both correction rate speeds and observed excess mortality (Figure 1).

## Supplementary Material
