# Supplementary material for: Hyponatremia Correction Rates and Mortality: Causality or Epiphenomenon?
Source: Kidney360. 2024 Mar 13;5(4):610–4. doi: 10.34067/KID.0000000000000414 (PMC11093530; doi:10.34067/KID.0000000000000414)
Supplement: Supplementary file 1 [file kidney360-5-610-s001.pdf]

ASN Journal Disclosure Form

As per ASN journal policy, I have disclosed any financial relationship or commitment held by myself and/or my spouse/partner in the past 36 months as included below. I have listed my Current Employer below to indicate there is a relationship requiring disclosure. If no relationship exists, my Current Employer is not listed.

H. Rondon Berrios reports the following:
Employer: University of Pittsburgh School of Medicine; University of Pittsburgh Medical Center; and Other Interests or Relationships: Editorial Board member, CJASN; Item-Writing Approval Committee member, ABIM Nephrology LKA; Remunerated for work as an expert witness in cases related to the treatment of hyponatremia by attorneys for both plaintiff and defense;.

I understand that the information above will be published within the journal article, if accepted, and that failure to comply and/or to accurately and completely report the potential financial conflicts of interest could lead to the following: 1) Prior to publication, article rejection, or 2) Post-publication, sanctions ranging from, but not limited to, issuing a correction, reporting the inaccurate information to the authors' institution, banning authors from submitting work to ASN journals for varying lengths of time, and/or retraction of the published work.

Name: Helbert Rondon Berrios
Manuscript ID: K360-2024-000102R2
Manuscript Title: Hyponatremia Correction Rates and Mortality: Causality or Epiphenomenon?
Date of Completion: February 27, 2024
Disclosure Updated Date: February 27, 2024

ASN Journal Disclosure Form

As per ASN journal policy, I have disclosed any financial relationship or commitment held by myself and/or my spouse/partner in the past 36 months as included below. I have listed my Current Employer below to indicate there is a relationship requiring disclosure. If no relationship exists, my Current Employer is not listed.

R. Sterns reports the following:
Employer: University of Rochester School of Medicine and Dentistry; Rochester General Hospital; and Other Interests or Relationships: 1) UpToDate Editor in Chief for Fluids and Electrolytes; 2) I have been remunerated for work as an expert witness in cases related to the treatment of hyponatremia. There is no company name; I have been contacted by attorneys for both plaintiff and defense.

I understand that the information above will be published within the journal article, if accepted, and that failure to comply and/or to accurately and completely report the potential financial conflicts of interest could lead to the following: 1) Prior to publication, article rejection, or 2) Post-publication, sanctions ranging from, but not limited to, issuing a correction, reporting the inaccurate information to the authors' institution, banning authors from submitting work to ASN journals for varying lengths of time, and/or retraction of the published work.

Name: Richard H. Sterns
Manuscript ID: K360-2024-000102R2
Manuscript Title: Hyponatremia Correction Rates and Mortality: Causality or Epiphenomenon?
Date of Completion: February 28, 2024
Disclosure Updated Date: June 5, 2023
